# Dual contrastive learning-based reconstruction for anomaly detection in attributed networks

**DOI:** 10.1371/journal.pone.0335135

**Published:** 2025-11-24

**Authors:** Hossein Rafieizadeh, Hadi Zare, Mohsen Ghassemi Parsa, Hocine Cherifi

**Affiliations:** 1 Department of Data Science and Technology, School of Intelligent Systems Engineering, University of Tehran, Tehran, Iran; 2 ICB UMR, CNRS, University of Burgundy (Université de Bourgogne), Dijon, France; The Hong Kong Polytechnic University, CHINA

## Abstract

Anomaly detection in attributed networks is critical for identifying threats such as financial fraud and intrusions across social, e-commerce, and cyber-physical domains. Existing graph-based methods face two limitations: (i) embedding-based approaches obscure fine-grained structural and attribute patterns, and (ii) reconstruction-based methods neglect cross-view discrepancies during training, leaving cross-view discrepancies underutilized. To address these gaps, we propose Dual Contrastive Learning-based Reconstruction (DCOR), a dual autoencoder with a shared Graph neural network (GNN) encoder that contrasts reconstructions (not embeddings) between original and augmented graph views. Instead of contrasting embeddings, DCOR reconstructs both adjacency and attributes for the original graph and for an augmented view, then contrasts the reconstructions across views. This preserves fine-grained, view-specific information and improves the fidelity of both structure and attributes. Across six benchmarks (Enron, Amazon, Facebook, Flickr, ACM, and Reddit), DCOR achieves the best Area Under the Receiver Operating Characteristic curve (AUROC) on six datasets. In comparison with the best-performing non-DCOR baseline across datasets, DCOR improves AUROC by 11.3% on average, with a maximum gain of 21.3% on Enron. On Amazon, ablating the reconstruction-level contrast (RLC) reduces AUROC by 25.5% relative to the model, underscoring the necessity of reconstruction-level contrastive learning. Code and datasets are publicly available at https://github.com/Hossein1998/DCOR-Graph-Anomaly-Detection.git.

## Introduction

Anomaly detection in attributed networks is crucial across various domains, including social media, e-commerce, finance, cybersecurity, and the Internet of Things (IoT). In these graphs, nodes carry attributes in addition to links, enabling rich modeling of behaviors and interactions. Detecting anomalies matters because they often correspond to security breaches, fraud, fake accounts, or sensor failures [1]. Prior estimates suggest that online payment fraud alone could amount to hundreds of billions of dollars over a few years [2], and fabricated influencer activity has incurred substantial losses annually [3]. Similar concerns arise in cyber-physical infrastructures, where anomalous nodes may indicate compromised devices or faulty sensors [4–7]. These examples motivate robust, scalable detectors for attributed graphs.

**Fig 1:**
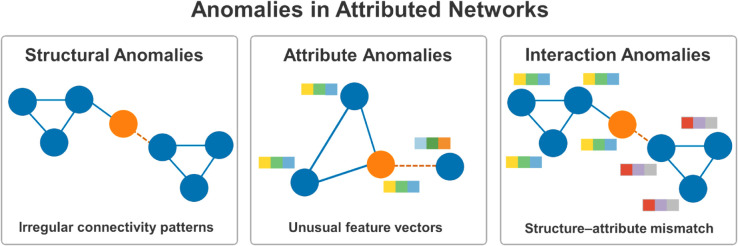
Anomalies in attributed networks. Structural anomaly: an unexpected inter-community bridge, where the orange node sits in the cut between two communities and forms a shortcut (dashed) edge to the right cluster. Attribute anomaly: the orange node’s feature vector (colored bars) deviates from those of its neighbors even though its connectivity looks normal. Interaction anomaly: a structure–attribute mismatch, where the orange node’s attributes align with the left community while its links embed it in the right community. Visual encoding: blue = normal nodes and edges; orange = anomalous node or edge; dashed orange edge = anomalous link; colored bars = node attributes.

Anomalies in attributed networks typically fall into three categories (Fig 1) [1,8,9]: (i) Structural: unexpectedly dense communities or cliques, spam links, or isolated nodes; (ii) Attribute: unusual or implausible feature values (e.g., abnormal transaction rates or incomplete profiles); (iii) Interaction: inconsistencies between structure and attributes (e.g., a highly connected merchant with suspicious transactional attributes). Accurately capturing these diverse anomaly types is nontrivial, especially when structural and attribute signals conflict or evolve dynamically.

**Table 1:** Abbreviations and notation.

Abbreviation	Definition
AEGIS	Adversarial Graph Differentiation Networks (method)
AnomalyDAE	Anomaly Detection Through a Dual Autoencoder (method)
AUROC	Area Under the Receiver Operating Characteristic curve
CONAD	Contrastive Attributed Network Anomaly Detection with Data Augmentation (method)
cuDNN	CUDA Deep Neural Network library
DCOR	Dual Contrastive Learning-based Reconstruction (proposed method)
DOMINANT	Deep Anomaly Detection on Attributed Networks (method)
ELU	Exponential Linear Unit
EMA	Exponential Moving Average
GAAN	Generative Adversarial Attributed Network (method)
GAD	Graph Anomaly Detection
GAT	Graph Attention Network
GNN	Graph Neural Network
GPU	Graphics Processing Unit
GraphSAINT	Graph Sampling-based Inductive Learning
InfoNCE	Information Noise-Contrastive Estimation
IoT	Internet of Things
KL	Kullback–Leibler Divergence
LLM	Large Language Model
LOF	Local Outlier Factor (method)
MFLOPs	Million Floating-Point Operations
MI	Mutual Information
ReLU	Rectified Linear Unit
RL	Reinforcement Learning
RLC	Reconstruction-level Contrast
SCAN	Structural Clustering Algorithm for Networks (method)
SSL	Self-supervised Learning
symKL	Symmetric KL Divergence

To effectively detect these diverse anomaly types, researchers have developed various approaches. Early detectors such as Local Outlier Factor (LOF) [10] compute density-based scores in an embedding space and implicitly assume that proximity captures normal behavior, treating anomalies as sparsely connected or isolated points. Structural Clustering Algorithm for Networks (SCAN) [11] clusters nodes by structural similarity and labels those not belonging to any structural cluster as outliers. While simple and efficient, these heuristics are ill-suited to high-dimensional attributes and cannot capture anomalies arising from subtle feature deviations or complex interactions. They also presuppose homophily (connected neighbors tend to have similar attributes), which fails in many modern applications (user–item graphs, anti-fraud networks, knowledge graphs). In such heterophilous graphs (connected neighbors tend to have dissimilar attributes), purely structural signals can mislead detectors: normal nodes near anomalous ones may be falsely flagged, and anomalous nodes surrounded by normal neighbors may be overlooked, leading to high false positives and negatives when attribute-driven or mixed anomalies are present [1,12].

To address these limitations, recent self-supervised learning (SSL) methods learn node representations without labeled anomalies by creating multiple graph views via augmentations (edge dropping, feature masking, subgraph sampling, or diffusion) and training encoders to maximize agreement for the same node across views, while minimizing agreement between different nodes. Early variants such as GraphCL (graph contrastive learning), GRACE (graph contrastive representation learning), and MVGRL (multi-view graph representation learning) [13–15] rely on random augmentations, whereas newer frameworks like GADAM (graph anomaly detection with adaptive message passing), AD-GCL (adversarial graph augmentation to improve graph contrastive learning), CONAD (contrastive attributed network anomaly detection with data augmentation), and UniGAD (unifying multi-level graph anomaly detection) [16–19] add adaptive message passing, adversarial view generation, and multi-level stitching. Despite its effectiveness, most graph contrastive pipelines use a narrow augmentation set (edge or node dropping, feature masking, subgraph sampling, or diffusion [20–22]), which can overlook key anomaly facets and sometimes produce unrealistic augmented views. We therefore adopt a richer, taxonomy-aligned augmentation suite that instantiates all three anomaly categories in Fig 1. Further details are provided in the proposed framework section.

**Fig 2:**
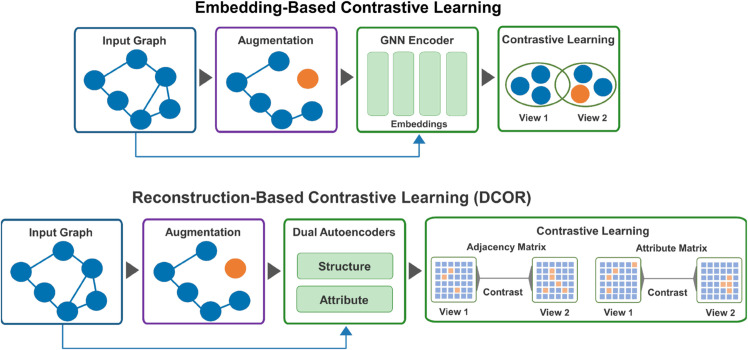
Embedding vs. reconstruction-level contrast (RLC). Top: the encoder consumes two inputs (the original graph and an augmented view) and contrasts their node embeddings in the embedding space. Bottom: DCOR uses dual autoencoders to reconstruct the adjacency and the attribute matrices for the original graph and the augmented view, then applies contrastive learning directly to the two sets of reconstructions, which preserves cross-view discrepancies that message passing may smooth out.

Nonetheless, in spite of strong downstream performance, these contrastive pipelines still compare compressed node embeddings produced by message passing. This compression can blur fine-grained anomaly cues, particularly in heterophilous graphs where anomalous nodes are largely surrounded by normal ones [23–26]. Moreover, message passing inherently smooths features across neighborhoods [27,28], leading to over-smoothing such as distinctive signals may be washed out [16,29] (see Fig 2).

Complementary to contrastive learning approaches, autoencoder- and graph neural network (GNN)-based architectures model graph data directly to address some of these limitations. DOMINANT (deep anomaly detection on attributed networks) [30], AnomalyDAE (anomaly detection through a dual autoencoder) [31], GAD-NR (graph anomaly detection via neighborhood reconstruction) [32]; CurvGAD (leveraging curvature for enhanced graph anomaly detection) [33], and MTGAE (mirror temporal graph autoencoder) [34] are representative examples in this category. These models score anomalies via reconstruction error but typically operate on a single view and therefore miss cross-view inconsistencies that often signal subtle anomalies. As a result, valuable discrepancies between reconstructions from different augmentations (e.g., a node well reconstructed in one view but poorly in another) remain underexplored.

These observations reveal two principal gaps:

**(A) Embedding-based methods** compare low-dimensional embeddings, erasing fine-grained anomaly signals (particularly in heterophilous networks where distinctive features become over-smoothed [26]).
**(B) Reconstruction-based methods** reconstruct the adjacency matrix *A* and nodal features *X* but do not compare reconstructions across augmented views, leaving cross-view discrepancies (and the opportunity to improve reconstruction quality) unexploited.

We introduce Dual Contrastive Learning-based Reconstruction (DCOR), a dual autoencoder framework trained with a reconstruction-level contrastive objective that directly compares reconstructed adjacency and attribute matrices across two augmented views. By contrasting reconstructions rather than embeddings, DCOR preserves view-specific cues, improves reconstruction fidelity, and enhances anomaly separability. Across six public benchmarks, DCOR attains best or competitive performance in terms of Area Under the Receiver Operating Characteristic curve (AUROC). We also adopt a taxonomy-aligned augmentation suite that augments the structure, attributes, and their interaction, providing comprehensive self-supervised signals.

Our contributions are threefold: (i) a reconstruction-level contrastive objective over decoded structure and attributes; (ii) a domain-informed augmentation suite that covers structural, attribute, and interaction anomalies; and (iii) a practical dual-autoencoder design with a shared GNN encoder.

This work extends our earlier conference paper [35] with substantial modifications, including new sections, additional experiments and evaluation metrics, as invited for journal publication.

This paper is organized as follows. The Related work section reviews graph-based anomaly detection, including traditional methods, autoencoder- and GNN-based models, and contrastive learning. The Proposed framework section introduces DCOR, detailing the dual-autoencoders, the reconstruction-level contrastive objective, and taxonomy-aligned augmentations. The Experimental results section describes datasets, evaluation metrics, and implementation, and reports empirical findings, ablations, and robustness analyses. The Discussion section examines limitations, practical considerations, and future directions. The Conclusion section summarizes the paper and highlights the key findings.

## Related work

This section reviews four strands: (i) traditional detectors, (ii) autoencoder- and GNN-based models, (iii) contrastive learning (including augmentation-oriented, adversarial, and RL-assisted variants), and (iv) domain applications. Table 2 provides a side-by-side summary of these approaches.

**Table 2:** Condensed summary of graph anomaly detection methods and their key strengths and limitations.

Category / Method	Pros	Cons
**Traditional**		
LOF [10]	Simple unsupervised density scoring	Sensitive to *k* and metric; repeated runs at scale are costly
SCAN [11]	Detects structural outliers from topology	Ignores attributes; fragile to noisy links
RADAR [36]	Joint structure–attribute modeling (low-rank + sparse with neighborhood regularization)	Expensive optimization on large graphs; assumptions often homophily-leaning
ANOMALOUS [37]	CUR-based selection + residual analysis	Cost grows with graph size and feature; may miss mixed anomalies
**Autoencoder and GNN**		
DOMINANT [30]	Joint reconstruction of topology (adjacency) and node attributes	Over-smoothing under heterophily; single-view (no cross-view contrast)
AnomalyDAE [31]	Dual decoders for structure and attributes	Heavy on very large graphs; single-view
GAD-NR [32]	Neighborhood reconstruction; often better than global matrix methods	Depends on neighborhood assumptions; may degrade under heterophily; single-view
CurvGAD [33] and MTGAE [34]	Higher-order geometry and multi-task heads	Extra overhead; single-view; no cross-view contrast
**Contrastive learning**		
GraphCL, GRACE, MVGRL [13–15]	Strong SSL via augmentations and view contrast	Embedding-level comparison; fine cues can vanish
CONAD [18]	Anomaly-aware augmentations; uses recon for scoring	Still compares embeddings; information loss possible
GCCAD [40], GAD-MSCL [41]	Cross/multi-scale contrast enriches semantics	Sensitive to augmentation and tuning; embedding limitations remain
EAGLE [42], CoModality [43]	Edge/modality-aware signals	Embedding-space comparison; modality quality critical
GADAM [16]	Adaptive message passing for anomalies	More complexity; still latent-space comparison
AD-GCL [17]	Adversarial hard views; strong backbones	Not GAD-specific; adversarial overhead
UniGAD [19]	Unified multi-level GAD (stitching etc.)	Complex pipeline; orthogonal to recon-level contrast
CoLA / multi-view subgraphs [47–50]	Captures local dependencies around nodes	Many subgraphs; runtime and memory costs.
**Hybrid and Robustness-Oriented Variants**		
AugAN [45]	Semi-supervised generalization with data augmentation	Requires labeled nodes; cross-entropy on embeddings
ARANE [53], GAAN [54]	Robustness or synthesis can help detection	Training complexity and stability concerns
SUGAR [52], RAND [51]	Pooling and selection highlight informative regions	Extra compute and tuning; scalability limits
AEGIS [58], AdvGraLog [56], RegraphGAN [55], SGAT-AE [57]	Inductive or domain-specific robustness	Hyperparameter + adversarial overhead; domain-specific

### Traditional anomaly detection in graphs

Classical methods rely on local density, structural similarity, or low-rank and sparse decompositions. LOF [10] assigns density-based outlier scores and is effective in many tabular settings. However, LOF is sensitive to the neighborhood size *k* and the choice of distance metric, and repeated runs at scale can be costly. SCAN [11] clusters nodes by structural similarity and flags non-members as outliers. By design, SCAN focuses on topology and ignores node attributes, which causes limited sensitivity to attribute-driven irregularities.

The second category of works relies on modeling attributed graphs via matrix-based formulations. Residual Analysis for Anomaly Detection in Attributed Networks (RADAR) [36] couples structure-attribute effects with a low-rank and sparse decomposition and neighborhood regularization. The smoothness assumptions of RADAR often align with homophily and can degrade under heterophily. ANOMALOUS (joint modeling for anomaly detection on attributed networks) [37] factors the reconstructed structure-attribute matrix via column–row decomposition (CUR) and scores residuals. Like other decomposition methods, its cost grows with graph size and feature dimensionality.

### Autoencoder and GNN-based approaches

These models reconstruct topology and attributes and typically score anomalies via reconstruction residuals. While effective on attributed graphs, they may suffer from over-smoothing under heterophily, and when trained in a single view, they overlook cross-view discrepancies. DOMINANT [30] jointly reconstructs *A* and *X* and mixes adjacency and feature errors to score anomalies; it often performs well. However, small reconstruction gaps can reduce sensitivity, and under strong heterophily, message passing may over-smooth distinctive cues [26,29]. AnomalyDAE [31] uses dual decoders to reconstruct adjacency and attributes, improving coverage of structural and attribute anomalies, yet training can be heavy on very large graphs, and it does not compare reconstructions across augmented views. GAD-NR [32] regularizes neighborhood reconstruction of structure and attributes to capture complex structural anomalies and often scales better than global matrix methods, but it depends on neighborhood assumptions and can degrade under pronounced heterophily or irregular local mixtures. CurvGAD [33] integrates discrete Ricci curvature to encode higher-order geometry at additional computational cost and still without cross-view comparison. MTGAE [34] is a multi-task graph autoencoder for topology and attributes (with temporal or auxiliary heads) and shares the trade-offs above: potential over-smoothing [26,29] and no explicit cross-view comparison.

### Contrastive learning for graph representation

Beyond GraphCL [13], frameworks such as GRACE [14] and MVGRL [15] construct augmented graph views via random edge and node dropping, feature masking, subgraph sampling, or diffusion, and maximize agreement of the same node across views using the InfoNCE loss (Information Noise-Contrastive Estimation) [38]. While effective for unsupervised representation learning, these pipelines compare node embeddings in embedding space, which can attenuate fine-grained structural or attribute cues due to message passing and over-smoothing, particularly on heterophilous graphs. More recent variants add adaptive or adversarial guidance to better align with anomaly patterns: GADAM [16] adapts message passing; AD-GCL crafts adversarial augmentations to produce harder views [17]; and UniGAD [19] unifies multi-level detection (e.g., graph stitching). Nevertheless, most pipelines still rely on a limited augmentation set and compare in embedding space, where over-smoothing and over-squashing [39] can blur fine-grained cues, especially under heterophily. Our approach targets both gaps by enforcing reconstruction-level contrast across views on topology and attributes.

A related line detects anomalies by comparing learned node representations, including CONAD. Representative examples include GCCAD (graph contrastive learning for anomaly detection) [40], GAD-MSCL (graph anomaly detection via multi-scale contrastive learning) [41], EAGLE (efficient contrastive-learning-based anomaly detector on graphs) [42], CoModality [43], and semi- and weakly supervised variants [44]. These methods inherit the strengths of contrastive learning yet remain vulnerable to information loss when embeddings are smoothed or when perturbations are subtle.

Recent surveys systematize a broad toolbox of graph augmentations, including edge perturbation (addition-removal-rewiring), node or edge dropping, feature masking or denoising, subgraph sampling, and diffusion-style transforms [20–22]. In practice, however, many graph anomaly detection (GAD) studies instantiate only a narrow subset (for example, random edge dropping or adding and simple feature masking), and overly aggressive or poorly matched perturbations can yield unrealistic structures or erase salient signals, thereby harming anomaly separability. In contrast, we adopt a broader, controlled suite with explicit budgets and topology and attribute constraints, covering structure-level patterns (clique injection, node isolation, random connections, inter- and intra-community rewiring or removal) and feature-level patterns (copying, scaling, masking) derived from domain knowledge.

### Hybrid and robustness-oriented variants

#### Augmentation-based semi-supervised variants.

AugAN (augmentation for anomaly and normal distributions) [45] tackles generalized graph anomaly detection in a semi-supervised setting. It expands the scarce labeled set of normal and anomalous nodes via data augmentation and adopts a tailored episodic training strategy so that the learned representations and classifier remain effective on both unseen subgraphs and entire graphs. NodeAug (node-parallel augmentation) [46] applies feature and edge augmentations to regularize semi-supervised node classifiers. In contrast, we use structural and feature augmentations solely to synthesize training views and train via reconstruction-level contrast without any ground-truth labels. The final anomaly scores are derived from reconstruction discrepancies.

**Reinforcement learning-assisted and adversarial variants.** Subgraph-centric approaches (e.g., CoLA (contrastive self-supervised learning framework for anomaly detection) [47]) form positive and negative pairs between a node and random-walk subgraphs from its neighborhood versus other nodes to capture local structure-attribute dependencies, but constructing many subgraphs incurs non-trivial cost such as multi-view and multi-scale methods [48–50] with scalability limits. RL-assisted methods surface informative structure either via neighborhood selection (RAND [51]) or mutual information (MI)-driven pooling (SUGAR [52]). Adversarial formulations include embedding regularization (ARANE (adversarially regularized attributed network embedding) [53]), data synthesis (GAAN (generative adversarial attributed network) [54]), and domain-specific generators (RegraphGAN (graph generative adversarial network for dynamic network anomaly detection) [55], AdvGraLog (graph-based log anomaly detection via adversarial training) [56], SGAT-AE (self-learning graph attention network autoencoder) [57]), and inductive anomaly-aware layers AEGIS (adversarial graph differentiation networks) [58] with better robustness. These variants broaden robustness but add training complexity, and many still operate at the embedding level, risking information loss.

### Anomaly detection in specialized domains

Graph-based anomaly detection is effective across multiple domains. In social networks, GNN-based detectors flag irregular user behavior and structural patterns such as fake profiles and misinformation spread [59]. In e-commerce, graph autoencoders uncover unusual co-purchase patterns and fraudulent transactions [60]. In IoT, modeling device-device interactions enables detection of sensor faults and malicious activities. Recent surveys review GNN- and AI-based IoT anomaly detection [61,62]. Financial networks benefit from large-scale graph benchmarks for fraud and risk detection [63]. In healthcare, graph analysis over patient-provider-claim relations has been used to detect anomalous or fraudulent behavior [64].

**Synthesis and positioning.** Traditional detectors (e.g., LOF) score local density and typically ignore joint modeling of structure and attributes [10]. Autoencoder- and GNN-based models (e.g., DOMINANT) jointly reconstruct *A* and *X* but are trained in a single view and can over-smooth signals [30]. Contrastive GAD (e.g., CONAD) compares augmented views in the embedding space, where fine-grained cues may be compressed [18]. RL and adversarial approaches (e.g., SUGAR) add complexity [52]. DCOR differs by addressing both gaps: it enforces reconstruction-level contrast across two augmented views with dual-autoencoders and a controlled augmentation suite, explicitly preserving cross-view discrepancies in *A* and *X*.

## Proposed framework

In this section, we formalize the task and present DCOR’s end-to-end pipeline. The overall structure of the proposed approach is given as,

- Employ domain-informed graph augmentations to induce realistic structural and attribute anomalies, including clique injection, node isolation, shortcuts, community-level changes, and feature copying, scaling, and masking.
- Use a sampling strategy to create mini-batch subgraphs maintaining alignment between original and augmented views for efficient, robust training.
- Implement a dual-autoencoder model with shared graph-attention encoder and separate reconstruction heads for adjacency and features, enabling fine-grained anomaly detection.
- Apply reconstruction-level contrastive loss to pull reconstructions close for normal nodes and enforce a learnable margin separation for augmented (anomalous) nodes in both modalities.
- Optimize a total loss combining reconstruction fidelity and contrastive separation, with adaptive margin to balance calibration and enhance anomaly differentiation.
- Define node-level anomaly scores as reconstruction discrepancies in structure and features, ranking nodes by deviation for anomaly detection as the output.

In the following, required definitions and notations are presented. Then, each of the steps is illustrated.

### Notations and definitions

Lets consider an attributed, undirected, simple graph denoted by G=(V,E) where *V* is the set of nodes and *E* is the set of edges between nodes $u_{i},u_{j}\in V$. On the representation level, the graph is presented as G=(A,X), where $A\in \{0,1{\}}^{n\times n}$ is the adjacency matrix ($A=A^{⊤}$, *A*_*ii*_ = 0) and $X\in {\mathbb{R}}^{n\times d}$ stacks the node features (*n* nodes, *d* features per node).

A graph augmentation is an operator Aug that maps (*A*,*X*) to a new view

$$(A^{\prime },X^{\prime })=\mathrm{Aug}(A,X)=(A+\Delta A,\;X+\Delta X),$$ (1)

where ΔA applies edge-level perturbations (adding or deleting edges) while preserving symmetry and no self-loops, and ΔX applies attribute-level perturbations (e.g., copying, scaling, or masking feature entries). For clarity, we use “augmentation” for the operator Aug that generates the view, and “perturbations” for the concrete changes (ΔA,ΔX) applied to (*A*,*X*).

Let V={1,…,n} denote the node index set. For each augmented view, we record the nodes whose structure or attributes are perturbed, yielding subsets $S_{A},S_{X}\subseteq V$ (which may overlap), and define binary indicators

$$s_{i}^{\left(A\right)}=𝕀\{i\in S_{A}\},\;s_{i}^{\left(X\right)}=𝕀\{i\in S_{X}\},\;i\in V.$$ (2)

Equivalently, $S_{A}=\{i:\|\Delta A_{i\cdot }{\|}_{0}>0\}$ and $S_{X}=\{i:\|\Delta X_{i\cdot }{\|}_{0}>0\}$, where $\|\;\cdot \;{\|}_{0}$ counts nonzero entries and, for any matrix *M*, $M_{i\cdot }$ denotes row *i*. The superscripts (*A*) and (*X*) indicate whether the perturbation arises from the structural or attribute side, respectively. Due to training the model in a self-supervised manner without accessing to ground-truth values, these indicators serve only to gate the training loss and are never used during inference.

### Graph data augmentation

**Fig 3:**
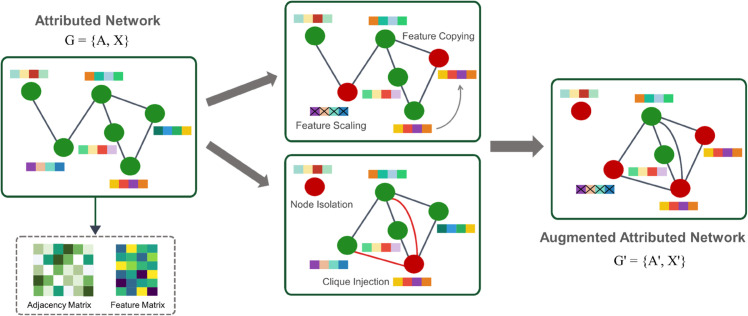
Structural and node-level augmentations for graph anomalies. Left: original attributed network *G*. Right: augmented attributed network $G^{\prime }$. Middle: augmentation methods: (1) feature copying (attribute mimicking across distant nodes); (2) feature scaling (multiplying or dividing continuous attributes); (3) node isolation (dropping all incident edges of selected nodes); (4) random shortcut connections and clique injection (adding shortcuts or small dense cliques across and within communities). Color coding: green nodes are normal; red nodes are augmented (selected for structure or feature augmentations; isolated nodes appear red with no incident edges); gray edges are original connections; orange or red edges indicate injected connections (random shortcuts or clique edges); solid feature bars are original attributes; cross-hatched bars mark augmented features; the thin gray curved arrow in the feature panel indicates attribute copying. Collectively, these augmentations induce structural, attribute, and interaction anomalies, creating cross-view discrepancies leveraged by our reconstruction-level contrast.

We employ domain-informed augmentations to synthesize realistic structural and attribute anomalies. Interaction mismatches arise implicitly when structure and attributes are augmented on different (possibly overlapping) node subsets. An overview of the two views is shown in Fig 3 (left: original graph *G*, right: augmented graph $G^{\prime }$).

On the structure side, several augmentations are employed including clique injection, node isolation, random shortcut edges, and community-level augmentations (inter-community bridging and intra-community edge removal). On the feature side, we apply feature copying (from another node), feature scaling, and feature masking. Optionally, light Gaussian noise is added to mimic measurement noise.

#### Sampling via GraphSAINT.

We adopt the GraphSAINT random-walk sampler [65] to construct mini-batch subgraphs. At each training step, a fresh subgraph is drawn from short random walks on the original graph, and the same node set is used to slice both the original and augmented views to maintain alignment. This resampling bounds memory usage and improves throughput while approximately preserving local connectivity. It also increases robustness by exposing the model to diverse, overlapping neighborhoods and by reducing brittleness to batch boundaries and occasional noisy or missing edges. Sampler settings are chosen to balance coverage and GPU (Graphics Processing Unit) budget.

### Structural graph augmentation

We inject controlled topological augmentations to mimic common anomaly patterns [20,21]. These augmentations are used only to generate augmented views for reconstruction-level contrast. This stage consists of four types of augmentation ways,

(i) Clique Injection,
(ii) Node Isolation,
(iii) Random Shortcut Edges,
(iv) Community-level augmentations containing inter-community bridging and intra-community edge removal.

#### Clique injection.

In social networks, tightly connected groups of anomalous nodes (cliques) often indicate coordinated malicious activities (e.g., fraud rings, botnets, organized misinformation). To reveal this behavior, a subset C⊆V is randomly selected and then every pair within *C* is connected to form a complete subgraph. Formally,

$$A_{ij}^{\prime }=A_{ji}^{\prime }=1\;(i\neq j,\;i,j\in C),\;A_{ii}^{\prime }=0,$$ (3)

where C⊆V with |C|=c, n=|V|, and $A^{\prime }\in \{0,1{\}}^{n\;\times \;n}$ denotes the augmented (binary) adjacency matrix (entries equal 1 when an edge is present and 0 otherwise); self-loops are disallowed and symmetry is preserved. For all other off-diagonal pairs (i,j)∉C×C, we keep the original connectivity, i.e., $A_{ij}^{\prime }=A_{ij}$. Typically c∈{2,…,n}. By adding these densely connected substructures, the model learns to recognize unusually dense local connectivity (Fig 3).

#### Node isolation.

Sometimes, anomalies such as compromised accounts or system failures can appear as users who become structurally isolated. To recognize this type of anomaly, we assign the isolation primitive to a random subset of nodes and delete all their incident edges, thereby zeroing the corresponding rows and columns in the augmented adjacency. This encourages the model to identify structural isolation as an anomalous connectivity pattern (Fig 3).

Let $S_{iso}\subseteq V$ be the nodes assigned isolation. We set

$$A_{ij}^{\prime }=0\;\text{whenever }i\in S_{iso}\text{ or }j\in S_{iso}.$$ (4)

For all $i,j\notin S_{iso}$, the original adjacency holds, i.e., $A_{ij}^{\prime }=A_{ij}$. We also set $A_{ii}^{\prime }=0$ and enforce symmetry as defined above.

#### Random shortcut edges.

We simulate unexpected links by adding a few shortcut edges between previously non-adjacent nodes. Additions are symmetric and exclude self-loops. Also, we avoid connecting to isolated nodes (Fig 3).

$$A_{ij}^{\prime }=A_{ij}\;\vee \;𝕀\;\left\{\;i\in S_{\text{add}},\;j\in J_{i}\;\right\},\;i<j;\;A_{ii}^{\prime }=0,$$ (5)

where $S_{\text{add}}\subseteq V$ specifies seed nodes for additions, each $J_{i}\subseteq \{\;j\in V\;⧵\;\{i\}:A_{ij}=0\;\}$ is a small non-neighbor set, and ∨ denotes logical OR.

#### Inter-community bridging.

Initially, a community partition $𝒫=\{{𝒞}_{1},\ldots ,{𝒞}_{K}\}$ of *V* is obtained by Louvain algorithm [66], where $C_{i},\;i=1,\cdots ,K$ denotes the communities, and *K* is the number of communities. For distinct communities ${𝒞}_{a}\neq {𝒞}_{b}$, we simulate unexpected cross-community ties by adding edges between non-neighbor pairs (measured w.r.t. *A*). Let


$$E_{cand}(a,b)=\{(u,v)\in {𝒞}_{a}\;\times \;{𝒞}_{b}:A_{uv}=0\},$$

$B\subseteq E_{cand}(a,b)$ is uniformly sampled without replacement with $|B|\leq k_{bridge}$ (a small addition budget). On augmentation side, set:

$$A_{uv}^{\prime }=1\;\;\text{for }(u,v)\in B,\;A^{\prime }=(A^{\prime }{)}^{⊤},\;\;A_{ii}^{\prime }=0.$$ (6)

To preserve isolation, additions are skipped incident to nodes in $S_{iso}$. This weakens community separation just enough to expose unusual cross-community interaction patterns.

#### Intra-community edge removal.

We first obtain a community partition (via the Louvain method [66]) and pick one community *C*. To simulate weakened internal cohesion, a small subset of edges is removed inside *C*:

$$A_{ij}^{\prime }=0\;\text{for }(i,j)\in J\subseteq \{(i,j):i<j,\;i,j\in C,\;A_{ij}=1\},$$ (7)

where *J* denotes the set of removed intra-community edges with $|J|\leq k_{rem}$ for a small budget $k_{rem}\in \mathbb{N}$. Symmetry and a zero diagonal properties are preserved by setting $A_{ji}^{\prime }=A_{ij}^{\prime }$ and $A_{ii}^{\prime }=0$.

### Node-level feature augmentation

Controlled feature augmentations are applied to imitate attribute-driven anomaly patterns [20,21]. These augmentations are just used to generate augmented views for reconstruction-level contrast. Three methods are applied: (i) Feature Copying, (ii) Feature Scaling, and (iii) Feature Masking.

#### Feature copying (Attribute Mimicking).

To induce attribute mismatches, for each node $i\in S_{X}$ we sample a candidate set $C_{i}\subseteq V\;⧵\;\{i\}$ of size s∈{1,…,|V|−1} uniformly without replacement, and copy the features from the farthest candidate in $\ell_{2}$:

$$k^{*}(i)=*{arg\;max}_{j\in C_{i}}\;\|X_{j}-X_{i}{\|}_{2}^{2},\;X_{i}^{\prime }=X_{k^{*}(i)}.$$ (8)

Ties in the *argmax are broken arbitrarily. Here, *V* is the node set, *S*_*X*_ the feature-side augmented nodes, *s* the pool size, *C*_*i*_ the candidate set for node *i*, $\|\;\cdot \;{\|}_{2}$ the Euclidean norm, *k* (*i*) the index of the farthest candidate, $X\in {\mathbb{R}}^{n\;\times \;d}$ the feature matrix, and $X_{i}^{\prime }$ the updated feature vector of node *i* (Fig 3).

#### Feature scaling.

To simulate magnitude shifts, for each node $i\in S_{X}$, its (continuous) feature vector is rescaled by a fixed factor α>1, randomly up or down,

$$X_{i}^{\prime }=\alpha^{\zeta_{i}}X_{i},\;\mathbb{P}(\zeta_{i}=1)=\mathbb{P}(\zeta_{i}=-1)=\frac{1}{2},$$ (9)

where *X*_*i*_ is the original feature vector of node *i*, $X_{i}^{\prime }$ the scaled one, *S*_*X*_ the set of nodes chosen for feature-side augmentation, α>1 the scale factor, and $\zeta_{i}\in \{+1,-1\}$ selects multiplication ( + 1) or division (–1) with equal probability. Only continuous features are scaled (Fig 3).

#### Feature masking.

Missing fields are simulated by zeroing a small random subset of feature dimensions for nodes on the feature side ($i\in S_{X}$). For node *i* with *d* features, sample $I_{i}\subseteq \{1,\ldots ,d\}$ uniformly without replacement with $|I_{i}|=\lfloor qd\rfloor$ (masking rate q∈(0,1)) and define the binary mask $m_{i}\in \{0,1{\}}^{d}$ by $(m_{i}{)}_{\ell }=0$ if $\ell \in I_{i}$, otherwise 1. Then

$$X_{i}^{\prime }=m_{i}⊙X_{i},$$ (10)

where *X*_*i*_ is the original feature vector of node *i*, $X_{i}^{\prime }$ the masked one, *S*_*X*_ the set of nodes chosen for feature-side augmentation, *d* the feature dimension, *q* the masking rate, *I*_*i*_ the zeroed indices, *m*_*i*_ the binary mask, ⊙ denotes element-wise multiplication, and ⌊·⌋ denotes the floor operator.

The structural and node-level feature augmentations illustrated in this section apply small, controlled modifications that mirror realistic anomaly patterns in social networks to enhance the model’s ability to detect subtle yet critical irregularities and improving robustness. We control augmentation budgets with per-dataset node-wise rates $\left(p_{A},p_{X}\right)$ and sample $S_{A},S_{X}$ independently (Bernoulli). Rates are tuned empirically via small ablation sweeps, and mean ± std are reported over three seeds. Formally, for each node *i*, $s_{i}^{\left(A\right)}\sim Bernoulli(p_{A})$ and $s_{i}^{\left(X\right)}\sim Bernoulli(p_{X})$, independently across nodes and across the two views, with $p_{A},p_{X}\in [0,1]$.

**Augmented-view diversity metrics.** To sanity-check that our augmented views are realistic yet diverse, we quantify the similarity between *G* and $G^{\prime }$ with scale-free metrics:

$$Edge\text{-}Jaccard=\frac{\left|E\cap E^{\prime }\right|}{\left|E\cup E^{\prime }\right|},$$ (11a)

$$Neigh\text{-}Jaccard=\frac{1}{n}\sum_{i=1}^{n}\frac{\left|{𝒩}_{i}\cap {𝒩}_{i}^{\prime }\right|}{\left|{𝒩}_{i}\cup {𝒩}_{i}^{\prime }\right|},$$ (11b)

$$Deg\text{-}symKL=\frac{1}{2}\;\left[KL(p_{\deg}\;\|\;q_{\deg})+KL(q_{\deg}\;\|\;p_{\deg})\right],$$ (11c)

$$Feat\text{-}cosine\text{-}mean=\frac{1}{n}\sum_{i=1}^{n}\frac{\langle x_{i},x_{i}^{\prime }\rangle }{\|x_{i}{\|}_{2}\;\|x_{i}^{\prime }{\|}_{2}+\varepsilon },$$ (11d)

where $E=\{(i,j):A_{ij}=1,\;i<j\}$ and $E^{\prime }=\{(i,j):A_{ij}^{\prime }=1,\;i<j\}$ are edge sets; ${𝒩}_{i}=\{j:A_{ij}=1\}$ and ${𝒩}_{i}^{\prime }=\{j:A_{ij}^{\prime }=1\}$ are neighbor sets. $p_{\deg}$ and $q_{\deg}$ denote the (normalized) empirical degree distributions of *A* and $A^{\prime }$, and KL(·‖·) is the Kullback–Leibler divergence. In (11d), *x*_*i*_ and $x_{i}^{\prime }$ are the *i*-th rows of *X* and $X^{\prime }$ (equivalently, $x_{i}\equiv X_{i}$ and $x_{i}^{\prime }\equiv X_{i}^{\prime }$), ⟨·,·⟩ is the dot product, $\|\;\cdot \;{\|}_{2}$ is the Euclidean norm, and ε>0 is a small constant.

### Contrastive learning framework

Contrastive learning distinguishes similar from dissimilar samples in a self-supervised manner. On graphs, comparing node embeddings across views can blur fine-grained structural or attribute cues, especially in heterophilous settings where message passing smooths distinctive signals [23–26,29]. In addition, reconstruction-based detectors usually operate on a single view and thus miss cross-view inconsistencies that are informative for anomalies. To address both issues, we contrast at the reconstruction level. Two views are considered,

$$G_{orig}=(A,X),\;G_{aug}=(A^{\prime },X^{\prime })=\mathrm{Aug}(A,X).$$ (12)

Then, a dual-branch reconstructor based on values of (12) is computed with shared weights ${ℛ}_{\theta }$ to obtain

$$(\hat{A},\hat{X})={ℛ}_{\theta }(A,X),\;({\hat{A}}^{\prime },{\hat{X}}^{\prime })={ℛ}_{\theta }(A^{\prime },X^{\prime }),$$ (13)

where ${ℛ}_{\theta }$ denotes the shared dual-branch reconstructor comprising structure and attribute autoencoders. *A*,*X* are the original adjacency and feature matrices, $A^{\prime },X^{\prime }$ are their augmented counterparts (hats indicate reconstructions).

During augmentation, we record which nodes were manipulated via the indicators in Eq (2). Our objective maintains cross-view reconstructions of non-augmented nodes close and applies an adaptive-margin penalty to augmented nodes: minimize structural and feature discrepancies for $s_{i}^{\left(A\right)}=0$ and $s_{i}^{\left(X\right)}=0$, and add a margin penalty when $s_{i}^{\left(A\right)}=1$ or $s_{i}^{\left(X\right)}=1$. “Non-augmented” and “augmented” are determined per node by $\left(s_{i}^{\left(A\right)},s_{i}^{\left(X\right)}\right)$, i.e., $s_{i}^{\left(A\right)}=0$ for structure and $s_{i}^{\left(X\right)}=0$ for features. The chosen discrepancy measures and the adaptive margin are detailed in the next subsection. $s_{i}^{\left(A\right)},s_{i}^{\left(X\right)}\in \{0,1\}$ are the structure- and feature-side augmentation indicators.

### Dual autoencoder model

**Fig 4:**
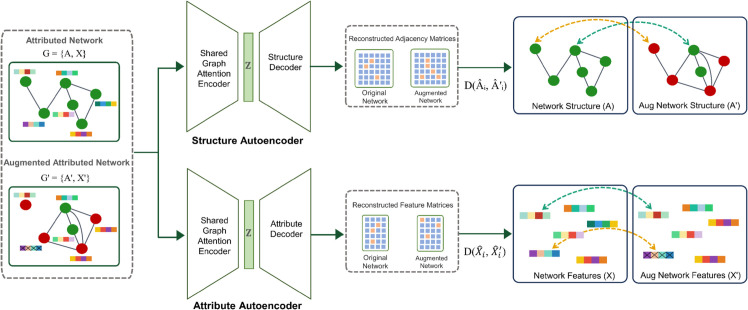
Dual autoencoder with reconstruction-level contrast. Left: an attributed network *G* and an augmented view $G^{\prime }$ produced by graph data augmentation. Middle: a shared graph-attention encoder yields node embeddings *Z*, which feed two decoders: a structure decoder reconstructing $\hat{A}$ and an attribute decoder reconstructing $\hat{X}$ for the two views, yielding $\left(\hat{A},\hat{X}\right)$ and $\left({\hat{A}}^{\prime },{\hat{X}}^{\prime }\right)$. Right: reconstruction-level contrast compares, for each node *i*, the reconstructions via $D({\hat{A}}_{i},{\hat{A}}_{i}^{\prime })$ and $D({\hat{X}}_{i},{\hat{X}}_{i}^{\prime })$; it minimizes *D* when $s_{i}^{\left(A\right)}=0$ and $s_{i}^{\left(X\right)}=0$, and enforces a learnable margin *m* when $s_{i}^{\left(A\right)}=1$ or $s_{i}^{\left(X\right)}=1$. Color coding: green nodes denote non-augmented nodes; red nodes denote augmented nodes; the dotted green arc indicates minimization of *D*; the dashed orange arc indicates margin enforcement; cross-hatched bars mark augmented features; gray edges are neutral; blue heatmaps depict reconstructed matrices.

Fig 4 illustrates our reconstruction-level contrast architecture for anomaly detection in attributed networks. The model uses a shared GAT-based encoder that produces node embeddings *Z*, followed by two reconstruction heads: a structural head that reconstructs the adjacency via an inner-product decoder, and an attribute head that reconstructs node attribute vectors via a linear decoder. The same weights are applied to both views $G_{orig}$ and $G_{aug}$, yielding $\left(\hat{A},\hat{X}\right)$ and $\left({\hat{A}}^{\prime },{\hat{X}}^{\prime }\right)$ that feed the contrastive objective. The rationale is specialization with sharing: structural and attribute anomalies have different signatures, so separate reconstruction heads preserve fine-grained cues in each modality, while a shared encoder ties the representations across views. By reconstructing both *A* and *X* for the original and augmented graphs and contrasting the two sets of reconstructions, the model highlights cross-view differences for augmented nodes, while maintaining consistency for non-augmented ones.

### Structure autoencoder

We use a shared GAT-style encoder to map node features to latent embeddings *Z*, which feed both decoders.

#### Encoder.

First project node features $X\in {\mathbb{R}}^{n\;\times \;d}$ to an intermediate representation

$$H=ReLU(XW_{s}+b_{s}),\;H\in {\mathbb{R}}^{n\;\times \;h},$$ (14)

with $W_{s}\in {\mathbb{R}}^{d\;\times \;h}$ and $b_{s}\in {\mathbb{R}}^{h}$ learnable. Then apply a GAT-style attention layer [67] to obtain node embeddings

$$Z=GAT(H,A),\;Z\in {\mathbb{R}}^{n\;\times \;d_{z}},$$ (15)

where *d*_*z*_ is the latent embedding dimension and *A* is the adjacency used for message passing after adding self-loops. Here *I* denotes the n×n identity matrix, and $N_{i}=\{\;j:A_{ij}>0\;\}$ is the neighbor set of node *i*.

The (additive) pre-softmax attention score between nodes *i* and *j* is

$$e_{ij}=a_{1}^{⊤}\;(W_{g}H_{i})+a_{2}^{⊤}\;(W_{g}H_{j}),$$ (16)

with learnable $W_{g}\in {\mathbb{R}}^{h\;\times \;d_{z}}$ and $a_{1},a_{2}\in {\mathbb{R}}^{d_{z}}$; here *H*_*i*_ denotes the *i*-th row of *H*.

Masked softmax over the neighbor set $N_{i}=\{\;j:A_{ij}>0\;\}$ gives

$$\alpha_{ij}=\frac{\exp(e_{ij})}{\sum_{k\in N_{i}}\exp(e_{ik})},$$ (17)

where *I* is the n×n identity matrix; this ensures $\sum_{j\in N_{i}}\alpha_{ij}=1$ for each *i*. Node embeddings then aggregate neighbor messages:

$$Z_{i}=ELU\left(\sum_{j\in N_{i}}\alpha_{ij}\;W_{g}H_{j}\right),$$ (18)

with ELU applied element-wise.

#### Decoder.

The adjacency matrix is reconstructed as,

$$\hat{A}=\sigma \left(ZZ^{⊤}\right),$$ (19)

where *Z* is the latent representation obtained from the encoder and *σ* denotes the sigmoid activation. This yields a symmetric edge-likelihood matrix $\hat{A}\in [0,1{]}^{n\;\times \;n}$. Following standard practice, we adopted the inner-product decoder used in graph autoencoders [68].

### Attribute autoencoder

This branch reconstructs node features by combining the shared node embeddings *Z* with a global attribute factor *F* learned from the current view’s features *X* (via $C=\frac{1}{n}X^{⊤}X$).

#### Encoder (global feature factorization).

We form a feature–feature summary matrix $C=\frac{1}{n}X^{⊤}X\in {\mathbb{R}}^{d\;\times \;d}$ and encode it as

$$H_{a}=ReLU(CW_{a1}+b_{a1}),\;H_{a}\in {\mathbb{R}}^{d\;\times \;h_{a}},$$ (20)

followed by

$$F=ReLU(H_{a}W_{a2}+b_{a2}),\;F\in {\mathbb{R}}^{d\;\times \;d_{z}},$$ (21)

where $W_{a1}\in {\mathbb{R}}^{d\;\times \;h_{a}}$, $W_{a2}\in {\mathbb{R}}^{h_{a}\;\times \;d_{z}}$, $b_{a1}\in {\mathbb{R}}^{h_{a}}$, and $b_{a2}\in {\mathbb{R}}^{d_{z}}$; here *h*_*a*_ is the attribute-encoder hidden width, and ReLU is applied element-wise. The same encoder is applied per view.

#### Decoder.

Attribute reconstruction is factorized as

$$\hat{X}=ReLU(ZF^{⊤}),\;\hat{X}\in {\mathbb{R}}^{n\;\times \;d},$$ (22)

where $Z\in {\mathbb{R}}^{n\;\times \;d_{z}}$ are the node embeddings from the shared GAT (graph attention network) encoder. For the augmented view, analogously have ${\hat{X}}^{\prime }=ReLU(Z^{\prime }(F^{\prime }{)}^{⊤})$.

Taken together, the shared encoder with dual decoders reconstructs the topology via $\hat{A}=\sigma (ZZ^{⊤})$ and the attributes via $\hat{X}=ReLU(ZF^{⊤})$ (and ${\hat{A}}^{\prime },{\hat{X}}^{\prime }$ for $G_{aug}$).

### Contrastive loss function

We use a reconstruction-level contrastive objective: given the original graph and an augmented view, the dual-autoencoders reconstruct both, and the loss pulls together reconstructions for non-augmented nodes while pushing apart those for augmented nodes via an adaptive margin. This focuses learning on structure and feature discrepancies introduced by augmentation and sharpens the separation between normal and anomalous nodes.

#### Structural contrastive loss.

The structural contrastive loss compares the reconstructed adjacency information from the original and augmented graph views. It encourages small reconstruction discrepancy for normal nodes and enforces at least a margin for nodes flagged as anomalous are depicted in the top-right panel of Fig 4:

$${ℒ}_{\text{struct}}=\frac{1}{n}\sum_{i=1}^{n}(𝕀\{s_{i}^{\left(A\right)}=0\}\;D({\hat{A}}_{i},{\hat{A}}_{i}^{\prime })\;+\;𝕀\{s_{i}^{\left(A\right)}=1\}\;\max\{0,\;m-D({\hat{A}}_{i},{\hat{A}}_{i}^{\prime })\}),$$ (23)

where $D(U,V)=\|U-V{\|}_{F}^{2}$ denotes the squared Frobenius norm, ${\hat{A}}_{i},{\hat{A}}_{i}^{\prime }$ are the *i*-th rows of the reconstructed adjacencies, 𝕀{·} is the indicator, $s_{i}^{\left(A\right)}\in \{0,1\}$ flags structural augmentation, and *m* > 0 is a learnable margin.

#### Attribute contrastive loss.

Analogously, the attribute contrastive loss compares reconstructed features across views (visualized in the bottom-right panel of Fig 4):

$${ℒ}_{\text{attr}}=\frac{1}{n}\sum_{i=1}^{n}(𝕀\{s_{i}^{\left(X\right)}=0\}\;D({\hat{X}}_{i},{\hat{X}}_{i}^{\prime })\;+\;𝕀\{s_{i}^{\left(X\right)}=1\}\;\max\{0,\;m-D({\hat{X}}_{i},{\hat{X}}_{i}^{\prime })\}),$$ (24)

where ${\hat{X}}_{i},{\hat{X}}_{i}^{\prime }$ are the *i*-th rows of the reconstructed feature matrices, and $s_{i}^{\left(X\right)}\in \{0,1\}$ flags feature-side augmentation; D(·,·) and *m* are as defined above.

**Positive and negative semantics.** We treat each modality independently. For structure, the pair $\left({\hat{A}}_{i},{\hat{A}}_{i}^{\prime }\right)$ is positive if $s_{i}^{\left(A\right)}=0$ (minimize $D({\hat{A}}_{i},{\hat{A}}_{i}^{\prime })$) and negative if $s_{i}^{\left(A\right)}=1$ (enforce the adaptive margin *m*) as in Eq (23). For attributes, the pair $\left({\hat{X}}_{i},{\hat{X}}_{i}^{\prime }\right)$ is positive if $s_{i}^{\left(X\right)}=0$ and negative if $s_{i}^{\left(X\right)}=1$ as in Eq (24). Thus, a node can be positive in one modality and negative in the other, depending on the applied augmentation. We do not construct inter-node negatives; contrast is performed intra-node across views only.

**Combined contrastive loss.** The reconstruction-level contrast ${ℒ}_{RLC}$ is defined as,

$${ℒ}_{RLC}\;=\;{ℒ}_{\text{struct}}\;+\;{ℒ}_{\text{attr}}.$$ (25)

#### Margin parameter.

The margin *m* > 0 in Eq (23) and Eq (24) sets the minimum discrepancy required between cross-view reconstructions for augmented nodes via the hinge term max{0,m−D(·,·)}. We treat *m* as a learnable scalar, and optimize it jointly with all model parameters by backpropagation, so the separation strength adapts to the data and augmentation difficulty. Unless stated otherwise, a single shared *m* is used for both structure and attributes; extending to modality-specific margins $m_{A},m_{X}\in \mathbb{R}>0$ is straightforward.

Why learn *m*? An adaptive margin reduces manual tuning, calibrates the loss across datasets and augmentation budgets, and mitigates under- or over-separation (collapsed positives or trivially large gaps). In contrast, most prior graph contrastive or reconstruction-based detectors rely on a fixed margin (or no margin at all), which can be miscalibrated across settings.

### Total loss function

A single objective is optimized to balance fidelity and separation by combining a reconstruction term and a reconstruction-level contrast term. Concretely, the total loss is a weighted sum of the reconstruction loss ${ℒ}_{rec}$ and the reconstruction-level contrast loss ${ℒ}_{RLC}$. Notably, ${ℒ}_{RLC}$ also acts as a cross-view regularizer: it enforces consistency of reconstructions for non-augmented nodes, thereby refining $\hat{A}$ and $\hat{X}$ and improving overall reconstruction fidelity.

$${ℒ}_{total}=\lambda_{rec}\;{ℒ}_{rec}+\lambda_{rlc}\;{ℒ}_{RLC},$$ (26)

where ${ℒ}_{RLC}={ℒ}_{\text{struct}}+{ℒ}_{\text{attr}}$ (with ${ℒ}_{\text{struct}}$ defined in Eq (23) and ${ℒ}_{\text{attr}}$ in Eq (24)), and $\lambda_{rec},\lambda_{rlc}>0$ control the trade-off between accurate reconstruction and discriminative separation.

**Reconstruction term.** We combine structural and attribute reconstruction errors with a modality weight λ∈[0,1]:

$${ℒ}_{rec}=\lambda \;\|A-\hat{A}{\|}_{F}+(1-\lambda )\;\|X-\hat{X}{\|}_{F},$$ (27)

where $\hat{A}=\sigma (ZZ^{⊤})$ and $\hat{X}=ReLU(ZF^{⊤})$. We use the Frobenius norm $\|\;\cdot \;{\|}_{F}$ (element-wise, without squaring) to linearly penalize reconstruction errors, complementing the squared discrepancies used in the contrastive terms.

**Discussion and settings.** Inside ${ℒ}_{rec}$, λ∈[0,1] interpolates between structural and attribute fidelity. At the outer level, $\lambda_{rec},\lambda_{rlc}>0$ control the trade-off between pure reconstruction and reconstruction-level contrast. Because $\|\;\cdot \;{\|}_{F}$ in ${ℒ}_{rec}$ and the squared discrepancies in ${ℒ}_{RLC}$ can have different scales, each term is normalized by its mini-batch moving average before weighting, then fixed coefficients are applied. This objective yields faithful reconstructions for non-augmented nodes and, via ${ℒ}_{RLC}$, enlarges cross-view discrepancies for augmentation-affected nodes, while regularizing reconstructions to remain view-consistent.

### Anomaly scoring

The proposed approach assign a node-level anomaly score from reconstruction discrepancies across both modalities. Intuitively, nodes whose reconstructed features or adjacency rows deviate substantially from the originals are more likely to be anomalous. The score combines structure- and attribute-side errors for each node $v_{i}$ as,

$$S(v_{i})=\alpha \;\|X_{i}-{\hat{X}}_{i}{\|}_{2}^{2}+(1-\alpha )\;\|A_{i}-{\hat{A}}_{i}{\|}_{2}^{2}.$$ (28)

Here, α∈[0,1] balances attribute vs. structural error. Nodes with larger $S(v_{i})$ are ranked as more anomalous. The dual-autoencoder is trained to reproduce prevalent (normal) structural and attribute patterns; augmented nodes are reconstructed poorly in the augmented view, and the reconstruction-level contrast further enlarges their cross-view discrepancies, yielding higher $S(v_{i})$.

## Experimental results

The proposed approach DCOR is evaluated on six standard attributed network datasets. This section details the datasets and the experimental setup. Then, the main results are presented along with training dynamics, ablations, augmentation-diversity checks, and robustness to anomaly prevalence shifts.

### Datasets and evaluation metric

Six widely used attributed network datasets are used in our study, which are given in Table 3 with important statistics, application domains, and anomaly rates. Enron [69] (an employee email communication network that captures interaction patterns and organizational relationships), Amazon [70] (a product co-purchase network in which nodes are products and edges indicate frequently co-purchased pairs, reflecting consumer buying behavior), Facebook [71] (a social network where nodes represent users and edges denote friendships (social ties)), Flickr [72] (an online photo-sharing network in which nodes are users and edges represent interactions among users), ACM [73] (an academic citation network whose nodes and edges capture publication entities and citation links; we follow the processed split commonly used for attributed graphs), and Reddit [74] (an online discussion forum network in which nodes represent users and edges reflect interactions such as replies or mentions; node attributes summarize content and metadata).

**Table 3:** Description of the datasets statistics and their anomaly ratios.

Dataset	Nodes	Edges	Attributes	Domain	Anomaly
Enron	13,533	176,987	18	Email network	0.04%
Amazon	1,418	3,695	21	Co-purchase network	1.97%
Facebook	4,039	88,234	576	Social network	9.9%
Flickr	7,575	239,738	12,407	Social network	5.9%
ACM	16,484	71,980	8,337	Citation network	3.6%
Reddit	10,984	168,016	64	Discussion forum	3.3%

**Evaluation metric.** Following prior work, we report the area under the receiver operating characteristic curve (AUROC) [75]. AUROC is threshold-free and rank-based, making it robust to severe class imbalance that is typical in anomaly detection.

### Implementation details

Our implementation uses Python and PyTorch [76] and runs on a single NVIDIA T4 GPU (Google Colab). We fix random seeds across Python, NumPy, and PyTorch, and enable deterministic settings in cuDNN (the CUDA Deep Neural Network library) for reproducibility. Raw graphs are loaded from MATLAB .mat files (adjacency, attributes, labels). Graphs are treated as undirected and unweighted: *A* is symmetrized, self-loops are added for message passing, and Louvain communities are computed on the unweighted graph without self-loops. The model input is the symmetrically normalized adjacency $\tilde{A}=D^{-1/2}(A+I)D^{-1/2}$, while (A+I) serves as the structure-reconstruction target. Here, *I* is the n×n identity and D=diag((A+I)1) is the degree matrix of (A+I). The architecture is a dual-autoencoder (GAT encoder; inner-product adjacency decoder; linear attribute decoder). Training uses Adam [77]. To scale to large graphs, we adopt GraphSAINT-style random-walk mini-batch sampling without reweighting; since our objective contrasts reconstructions across two views on matched node sets, we avoid estimator reweighting and accept the mild sampling bias for efficiency.

**Complexity and runtime.** Let *n* be the number of nodes, |E| edges, *d* input features, and *d*_*z*_ the embedding size. Message passing with sparse ops is $O(|E|\;d_{z})$. The inner-product decoder involves forming (sub)matrices like $ZZ^{⊤}$ at $O(n^{2}d_{z})$ if materialized. Parameter memory depends on layer sizes (e.g., $O(d\;h+h\;d_{z}+d_{z}^{2})$) and is independent of *n*; the dominant memory terms are activations $O(n\;d_{z})$ and any explicit reconstruction buffers for $\hat{A}$ (up to *O*(*n*^2^) if a full matrix is stored). On Amazon (n=1418, d=21, *d*_*z*_ = 128), we measured ∼190k parameters (∼0.7 MB), ∼265 MFLOPs (million floating-point operations) per forward pass, and ∼3 ms inference per 1k nodes; wall-clock scales roughly linearly with the number of epochs.

**Comparative note (vs. SOTA).** Training DCOR is ≈2× costlier than single-view reconstructors (e.g., DOMINANT [30], AnomalyDAE [31]) because each training step involves encoding and decoding two views. Unlike common InfoNCE-style pipelines (e.g., CONAD [18]), it typically does not build dense B×B similarity matrices or maintain large negative banks; and unlike adversarial schemes (e.g., GAAN [54]), it avoids generator and discriminator updates. With GraphSAINT subgraph sampling [65], the inner-product decoder’s *O*(*n*^2^) pairwise scoring reduces to $O(B^{2}d_{z})$ per mini-batch (full-pair scoring), where *B* is the number of nodes in the sampled subgraph (mini-batch size). Runtime memory is dominated by node and edge activations (and any optional $\hat{A}$ buffers). In inference, DCOR is single-pass (no augmentation or contrast), yielding a runtime comparable to DOMINANT and AnomalyDAE.

### Anomaly detection performance

We compare against strong baselines on six datasets and report AUROC (higher is better) in Table 4. DCOR attains the best AUROC on all six datasets.

**Table 4:** Anomaly detection performance (AUROC). Best per column in bold.

Method	Enron	Amazon	Facebook	Flickr	ACM	Reddit
LOF	0.581	0.510	0.522	0.661	0.473	0.412
DOMINANT	0.716	0.592	0.554	0.749	0.749	0.577
AEGIS	0.602	0.556	0.659	0.765	0.626	0.534
GAAN	0.731	0.651	0.712	0.746	0.727	0.568
AnomalyDAE	0.552	0.611	0.741	0.694	0.778	0.431
CONAD	0.731	0.635	0.863	0.782	0.762	0.561
**DCOR**	**0.887**	**0.795**	**0.892**	**0.822**	**0.846**	**0.617**

**Analysis.** Relative to the strongest non-DCOR baseline per dataset, absolute AUROC gains are  + 15.6 percentage points on Enron,  + 14.4 on Amazon,  + 2.9 on Facebook,  + 4.0 on Flickr,  + 6.8 on ACM, and  + 4.0 on Reddit (avg.  + 8.0 pp). In relative terms, these correspond to +21.34%, +22.12%, +3.36%, +5.12%, +8.74%, and +6.93% improvements, averaging +11.27% (+10.89% micro-averaged).

**Fig 5:**
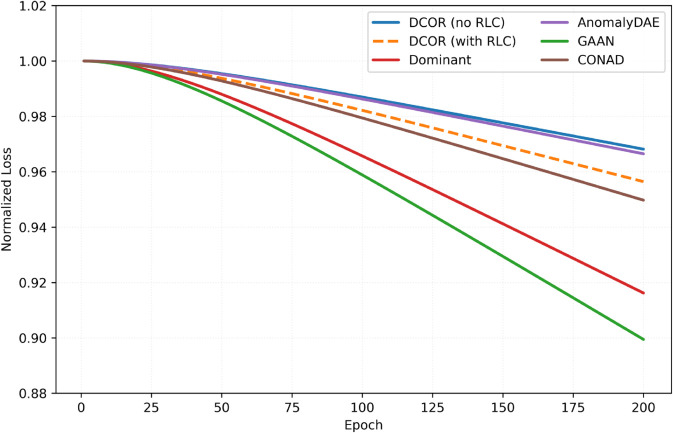
Normalized training loss vs. baselines (Facebook). DCOR reports both reconstruction-only and total (with RLC); baselines report reconstruction-only. Each curve is normalized as in Eq (29) by dividing by its epoch-1 value and EMA-smoothed (exponential moving average) with β=0.97, where the EMA is computed as $y^{\left(e\right)}=\beta \;y^{\left(e-1\right)}\;+\;(1\;-\;\beta )\;{\overset{\sim }{ℒ}}^{\left(e\right)}$ with $y^{\left(1\right)}={\overset{\sim }{ℒ}}^{\left(1\right)}$. This normalization enables fair visual comparison across methods with different objectives and scales; the plot therefore emphasizes relative convergence trends (shape and stability) rather than raw magnitudes. Consistent with DCOR’s design, RLC regularizes late-phase training: the reconstruction curve decreases more conservatively than methods that minimize reconstruction alone, while the total objective continues to decrease.

Beyond final AUROC, training dynamics are examined. Normalized losses over epochs are plotted, where each curve is divided by its own value at the first epoch to remove scale effects (Eq (29)). For DCOR, we reported both the reconstruction term and the total objective (with RLC); for baselines we reported the reconstruction-only term (Fig 5).

$${\overset{\sim }{ℒ}}^{\left(e\right)}=\frac{{ℒ}^{\left(e\right)}}{{ℒ}^{\left(1\right)}},\;e\in \{1,\ldots ,E\},$$ (29)

where ${ℒ}^{\left(e\right)}$ denotes the per-epoch loss and *E* is the number of epochs.

### Ablation study

We ablate three components on Amazon: (i) structural augmentation, (ii) feature augmentation, and (iii) the reconstruction-level contrast (RLC). Each variant removes exactly one component; the encoder and decoders, schedule, and all other hyperparameters are fixed. Results are summarized in Table 5.

**Table 5:** Ablation on Amazon. *Δ* is the signed difference vs. the full model (Δ=Variant−Full; negative indicates a drop).

Variant	AUROC	Δ
Full DCOR (ours)	**0.795**	—
w/o structural augmentation (feature only)	0.712	–0.083
w/o feature augmentation (structural only)	0.673	–0.122
w/o RLC (no reconstruction-level contrast)	0.592	–0.203

**Fig 6:**
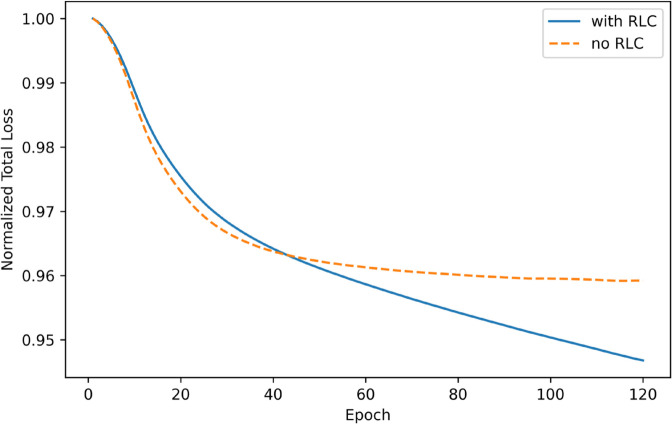
Normalized total loss on the Facebook dataset (DCOR with and without RLC). To enable a fair visual comparison of training dynamics, each curve is normalized as in Eq (29) by dividing by its epoch-1 value and EMA-smoothed (exponential moving average) with β=0.9. The EMA is computed as $y^{\left(e\right)}=\beta \;y^{\left(e-1\right)}+(1-\beta )\;{\overset{\sim }{ℒ}}^{\left(e\right)}$ with $y^{\left(1\right)}={\overset{\sim }{ℒ}}^{\left(1\right)}$. This normalization emphasizes relative convergence behavior (shape and stability) rather than raw magnitudes: with RLC, the total objective continues to decrease in late epochs, whereas without RLC it plateaus, consistent with the ablation trends in Table 5.

To visualize how RLC affects optimization, the total objective is tracked ${ℒ}_{\text{total}}=\lambda_{rec}{ℒ}_{rec}+\lambda_{rlc}{ℒ}_{RLC}$ over epochs and report a scale-free version normalized as in Eq (29) (Fig 6).

**Augmentation diversity.** Table 6 reports diversity metrics on representative datasets. Structural diversity is moderate (${div}_{edge}\;\approx \;0.24$–0.31), while the symmetric Kullback–Leibler (KL) on degrees remains small (≤0.06), indicating local edge perturbations without global distortion. Feature-level diversity differs by dataset: Facebook shows small changes ($1-c_{feat}\;\approx \;0.057$), whereas Flickr exhibits stronger shifts (sparser bag-of-tags). DCOR’s reconstruction-level contrast is trained to be invariant to such moderate view differences while preserving anomaly separability.

**Table 6:** Diversity of augmented graphs. Similarities (↑ larger = more overlap) and complementary diversities (↑ larger = more diverse). Lower is better for $D_{deg}$.

Dataset	$J_{edge}$ (↑)	$J_{neigh}$ (↑)	$D_{deg}$ (↓)	$c_{feat}$ (↑)	${div}_{edge}$ (↑)	${div}_{neigh}$ (↑)
Facebook	0.755	0.702	0.061	0.943	0.245	0.298
Flickr	0.765	0.690	0.014	0.158	0.235	0.310

As summarized in Table 5, removing RLC yields the largest AUROC drop (0.203). Using only feature augmentation reduces AUROC by 0.083, and using only structural augmentation reduces it by 0.122. On Amazon, feature-only outperforms structural-only by 0.039 (0.712 vs. 0.673), indicating a stronger self-supervised signal from attribute augmentations.

Notation. $J_{edge}$ and $J_{neigh}$ correspond to Edge-Jaccard and Neigh-Jaccard in Eq (11a) and Eq (11b); $D_{deg}$ equals Deg-symKL in Eq (11c); $c_{feat}$ equals Feat-cosine-mean in Eq (11d); ${div}_{edge}=1-J_{edge}$ and ${div}_{neigh}=1-J_{neigh}$.

### Robustness to varying anomaly ratios

To stress-test the robustness of our method to variations in anomaly prevalence, we keep the training procedure unchanged and vary, only during evaluation, the fractions of labeled structural and feature anomalies, using the same augmentation-based labeling protocol described in Subsection Graph data augmentation. On Enron, a mild setting (20% structural, 10% feature) yields an AUROC of 0.783, and a moderate setting (30%, 20%) yields 0.747. On Flickr, a mild setting (10%, 10%) yields 0.822, and a moderate setting (30%, 40%) yields 0.815. These results indicate that the ranking performance of our approach remains largely invariant under moderate shifts in anomaly prevalence.

## Discussion

This section summarizes the paper’s scientific contributions, highlights open challenges observed in practice, discusses the limitations and practical considerations of DCOR, and outlines future research directions that are closely aligned with our reconstruction-level contrast framework. The objective is to provide a transparent perspective on what DCOR accomplishes, where it faces challenges, and how it can be further extended.

### Contributions

The key contributions of this work are summarized, ensuring consistency with our formulation and experimental findings.

- **Reconstruction-level contrast (RLC) on decoded structure and attributes.** Instead of contrasting embeddings, DCOR performs contrastive learning directly on the reconstructions across two views, directly on $\hat{A}$ and $\hat{X}$ (Eq 23 to Eq 26), as illustrated in Fig 2. This preserves cross-view discrepancies that message passing may smooth out and improves anomaly separability.
- **Domain-informed augmentation suite.** We design a comprehensive and domain-informed augmentation suite that integrates both structural and attribute-level transformations. On the structural side, we employ techniques such as clique injection, node isolation, inter-community bridging, and intra-community edge removal. On the attribute side, we utilize feature copying, scaling, and masking to enrich feature diversity. This carefully controlled augmentation strategy provides self-supervised signals covering all three major anomaly taxonomies (structural, attribute, and interaction anomalies) while ensuring that the generated views remain realistic and faithful to the underlying graph semantics.
- **Learnable adaptive margin.** A positive, learnable margin are considered within the hinge terms of the reconstruction-level contrast loss. This adaptive margin automatically calibrates the separation strength across different datasets and augmentation budgets, thereby reducing the need for manual tuning and improving the robustness of the framework.
- **Scalable training with GraphSAINT.** We leverage GraphSAINT to enable scalable training through random-walk-based mini-batches with matched node sets across views. This strategy bounds memory consumption, preserves local connectivity, and stabilizes the reconstruction-level contrast during training.
- **Empirical validation across six benchmarks.** Extensive experiments on six real-world benchmarks revealed that DCOR outperforms state-of-the-art competitors in terms of AUROC (Table 4).

### Challenges

Extending the framework to million-node graphs remains challenging due to computational and memory constraints, even with efficient sampling strategies such as GraphSAINT. While the dual-autoencoder architecture is effective, it introduces additional training overhead. Furthermore, balancing the reconstruction and contrastive objectives requires careful tuning, as over-weighting either objective can degrade overall anomaly detection performance. Another practical challenge lies in selecting a compact yet representative set of anomaly scenarios for augmentation. In this study, we prioritized patterns most likely to occur in practice, including structural, attribute, and structure-attribute mismatches, consistent with established taxonomies [78–80]. This choice trades off some diversity for greater realism, a decision supported by our ablation study (Table 5). Finally, although DCOR effectively captures subtle irregularities, distinguishing true anomalies from naturally occurring network dynamics such as community evolution or legitimate attribute updates remains difficult. Prior work in dynamic community detection and temporal graph anomaly analysis highlights the prevalence of such phenomena [9,81,82]. Incorporating temporal information, domain-specific constraints, and post hoc validation pipelines may help mitigate false positives and enhance robustness in real-world deployments.

### Limitations

Although the proposed graph augmentation suite is designed to capture the three principal anomaly categories in graphs (structural, attribute, and interaction), it cannot fully encompass the diversity of real-world scenarios. Rare or domain-specific anomalies may fall outside this augmentation design space. Since DCOR relies on realistic augmented views, misaligned or overly aggressive augmentations can generate implausible structures or provide insufficient contrast between normal and anomalous nodes, thereby reducing detection accuracy. To mitigate this risk, we adopt a taxonomy-guided set of augmentations and examine sensitivity to augmentation budgets; nevertheless, broader and domain-adapted augmentation strategies remain necessary.

Another limitation pertains to the learnable margin in the contrastive loss. While the adaptive margin is intended to enhance separation, it can introduce training instabilities during early epochs if it adapts too rapidly or too slowly relative to batch difficulty. Achieving stable convergence therefore benefits from safeguards such as careful initialization, mild regularization, explicit lower and upper bounds on the margin, a brief warm-up phase, and gradient clipping.

### Future work

In future work, we aim to explore LLM (large language model)-guided, semantically coherent graph augmentations that produce context-aware modifications while preserving the underlying graph statistics and attribute semantics. These augmented views will be integrated into our dual-autoencoder contrastive framework to improve the detection of subtle, domain-specific anomalies.

We plan to perform comprehensive evaluations on datasets including Enron, Amazon, Flickr, and Facebook, with the goal of achieving higher AUROC scores. Furthermore, we intend to extend our approach to dynamic graphs and knowledge-rich networks, enabling more robust and temporally aware anomaly detection.

## Conclusion

DCOR introduces a novel paradigm for anomaly detection by contrasting reconstructed structures and attributes (rather than embeddings) across augmented graph views. This design preserves fine-grained, view-specific cues and significantly enhances the fidelity of both structural $\hat{A}$ and attribute $\hat{X}$ reconstructions, leading to superior anomaly separation.

Across six diverse benchmarks–including social, e-commerce, and academic networks–DCOR establishes new state-of-the-art results, achieving the highest AUROC on six datasets. It outperforms the strongest prior baseline by 11.3% on average, with a peak gain of 21.3% on Enron. ablation studies validate the method’s robustness: removing the reconstruction-level contrast causes a 25.5% AUROC drop on Amazon. These findings underscore the critical synergy between reconstruction-level contrast and complementary augmentations.

The effectiveness of DCOR can be attributed to four main aspects: the use of reconstruction-level contrast on decoded structure and attributes, a domain-informed augmentation suite that covers structural, attribute, and interaction patterns, a learnable margin that adapts the separation strength, and a dual-autoencoder architecture with a shared GAT encoder trained with GraphSAINT sampling for scalability.

Future work will extend DCOR to heterogeneous and dynamic graphs (e.g., temporal fraud networks) and optimize decoders for web-scale deployments, leveraging LLM-guided augmentations to handle complex data distributions.
